# Sleep duration and mental health in middle-aged and older adults: a study on gender differences

**DOI:** 10.3389/fpsyt.2025.1600300

**Published:** 2025-08-20

**Authors:** Jie Meng, Dan Li, Feng Sun, Lingling Tang, Jing Wang, Hui Li

**Affiliations:** ^1^ School of Health Services Management, Anhui Medical University, Hefei, China; ^2^ Hefei Center for Disease Control and Prevention, Hefei, China; ^3^ School of Public Health, Tongji Medical College, Huazhong University of Science and Technology, Wuhan, China; ^4^ Institute of Hospital Management, Anhui Medical University, Hefei, China

**Keywords:** sleep duration, mental health, middle-aged and older adults, gender differences, cross-sectional study

## Abstract

**Background:**

As the global population ages, mental health issues among middle-aged and older adults have become an important public health concern. Although the impact of sleep disorders on mental health is widely recognised, the specific mechanisms underlying the gender differences in mental health among middle-aged and older adults due to differences in sleep duration remain unclear. This study aims to explore the impact of sleep duration on mental health differences between middle-aged and older adults of different genders, providing key mechanistic evidence for understanding gender inequality in mental health among middle-aged and older adults.

**Methods:**

A total of 5 743 middle-aged and older people aged 45 years and above were surveyed. The 10-item Kessler Psychological Distress Scale (Kessler10) was used to evaluate mental health status. A self-designed questionnaire was used to collect information on participants’ socio-demographic characteristics, physical health status, and daily lifestyles. A multiple linear regression model was adopted to analyze the effect of sleep duration on mental health, and then the Oaxaca-Blinder decomposition for linear model was used to further explore the effect of sleep duration on gender differences in mental health.

**Results:**

The scores of mental health scale of male and female participants were 12.16(4.19) and 12.53(4.31), respectively. The regression result showed that sleep duration had a significant impact on the mental health status of different gender participants, with a stronger effect observed in females. Furthermore, the Oaxaca-Blinder decomposition indicated that approximately 20.57% of the mental health differences could be attributed to gender differences in sleep duration.

**Conclusions:**

Female middle-aged and older residents have poorer mental health compared to male residents. Moreover, the difference in sleep duration is an important component of the mental health gap between middle-aged and older people of different genders. These findings highlight the importance of improving the sleep quality of female residents in alleviating mental health issues among middle-aged and older adults, providing scientific evidence for the development of more gender-sensitive intervention strategies.

## Highlights

- The correlation between sleep duration and the mental health of middle-aged and older women is stronger than that of men.
- Gender differences in sleep duration could explain about 20.57% of mental health differences.
- Improving the sleep status of female is crucial to alleviate mental health problems in middle-aged and older adults.

## 1 Introduction

Population aging is a common trend in social development worldwide in the 21st century, and China is no exception. In 2020, the population aged 65 and above in China reached 190 million, accounting for 13.50% of the total population (1). Faced with rapid changes in social roles and status, older people are prone to mental problem. According to the life course theory, mental health is a comprehensive state that individuals presents throughout their entire lifespan by adapting and responding to changes in internal and external environments (2). Many health problems faced during old age can be prevented or delayed by early health behaviours. The middle age period is a critical stage in the transition from youth to old age. Negative life events such as physical function deterioration and retirement that individuals face after the age of 45 can not only cause fluctuations in mental health during middle age, but also extend to old age. Therefore, it is crucial to thoroughly investigate the factors affecting the mental health of middle-aged and older adults and develop intervention strategies.

Sleep duration, as one of the key indicators of sleep quality, is closely related to an individual’s mental health status (3–5). A team from Fudan University conducted a modelling analysis of sleep duration and mental health data from nearly 500,000 middle-aged and older adults (aged 38-73) and found that sleep duration has a significant non-linear association with mental health issues such as depression and anxiety. Both sleeping less than or more than 7 hours per night increases the risk of mental health issues (3). A meta-analysis by Scott et al. (6) of 65 randomised controlled trials showed that interventions such as appropriately controlling sleep duration can alleviate negative emotions like depression and anxiety. Notably, the association between sleep duration and mental health exhibits significant gender heterogeneity (7–10). Compared to men, women have shorter sleep duration and lower sleep efficiency, and women with sleep disorders have a higher comorbidity rate with anxiety and depression (9). A longitudinal study of Canadian adolescents found that chronic sleep deprivation (less than 8 hours per week) increases the risk of depression by 40% in young women, while no significant association was observed in men (10). Although the gender differences in the impact of sleep on mental health have garnered attention, most studies have focused on the general population or single-gender groups, lacking systematic gender comparisons for middle-aged and older adults. Additionally, few studies have quantified the specific contributions of sleep factors in gender differences, severely limiting the ability to develop precise intervention strategies tailored to gender characteristics.

Therefore, this study uses residents of Hefei City in central China as an example to explore whether there is a significant association between sleep duration and the mental health status of middle-aged and older people, and employs the Oaxaca-Blinder decomposition method to quantify the specific weight of sleep duration as a factor in the overall gender differences in mental health among middle-aged and older adults. Through methodological innovation, this study seeks to reveal the explanatory power of sleep factors in gender differences, providing scientific evidence for the development of gender-specific sleep intervention strategies to improve the mental health and quality of life in later years among middle-aged and older adults.

## 2 Methods

### 2.1 Study population and sampling

This study adopted a cross-sectional research design to conduct a questionnaire survey of participants. We focused on middle-aged and older adults in Hefei. Hefei is the capital city of Anhui Province, and located in central China. In 2020, 12.0% of its total population was aged 65 and over in Hefei, which was at the average level in China (1, 11). The sample size of this survey was calculated by using the formula: $n=z^{2}p\left(1-p\right)/d^{2}$ , where *n* is the sample size, *p* is the prevalence of the research target, *z* is the normal deviation (1.96), and *d* is the allowable error (*d*=0.1*p*). Literature showed that the prevalence of depressive symptoms among Chinese residents ranged from 23.0%-38.2% (12, 13). We took the median and set the 95% confidence interval to calculate the sample size of 872. To prevent respondents from randomly selecting answers, we included three quality control questions at different points in the electronic questionnaire and emphasised to participants throughout the survey that their answers were anonymous and that there were no right or wrong answers. If any of these questions were answered incorrectly, the Questionnaire Star platform marked the questionnaire as invalid. Taking into account non-responses and incomplete questionnaires (approximately 15%), at least 1,026 participants should be surveyed in each area.

By using the multi-stage stratified cluster sampling method, from August 1, 2021 to December 31, 2021, we randomly selected two counties (Changfeng and Feidong) and three municipal districts (Baohe, Luyang and Shushan) among the nine administrative districts in Hefei City as the monitoring area to carry out the survey. Five towns or streets were selected for each monitoring area, and one administrative village or neighbourhood committee was selected from each town or street. Therefore, a minimum of 206 samples should be surveyed in each administrative village or neighbourhood committee (1 026/5 = 205.2). This study relied on the staff of each township or street community health service station to conduct the survey at each centre simultaneously. To ensure the stability of the study results, we collected as large a sample size as possible. The survey was closed when the minimum sample size of 206 people was reached in each community health service station. In the end, a total of 5 743 people aged 45 and above were surveyed.

### 2.2 Measures

#### 2.2.1 Dependent variables

Mental health status was measured by using the 10-item Kessler Psychological Distress Scale (K10), a brief psychological status scale developed by Kessler et al. at the University of Michigan in 1994 (14). The scale consists of 10 self-report items and measures the frequency of nonspecific mental distress in the past month (e.g., “How often did you feel tired out for no good reason during the last 30 days?”). Using a 5-point Likert scale to score (1= none of the time, 2=occasionally, 3=some of the time, 4=most of the time, 5=all of the time). Thus, the total scores of K10 range from 10 to 50, with higher scores indicating poorer mental health.

#### 2.2.2 Independent variables

The participants’ sleep duration was assessed by setting a question: “How many hours of sleep did you get each day in the last month? “ According to the recommendations of the National Sleep Foundation, this study classified sleep duration into three categories: “<7h”, “7-8h” and “>8h” (15).

#### 2.2.3 Control variables

We selected three types of control variables related to mental health. The first type was the socio-demographic characteristics of middle-aged and older people, including age(<65 years, ≥65 years), marital status (unmarried/widowed/divorced/separated, married) and education level (≤6 years, >6 years). The second type was physical health status, including the prevalence of major chronic diseases such as hypertension, diabetes mellitus (yes, no), hyperlipidemia (yes, no) and self-reported health. Hypertension was defined as mean systolic blood pressure(SBP) ≥140 mmHg or diastolic blood pressure(DBP) ≥90 mmHg, or self-reported use of an antihypertensive drug in the past 2 weeks (16). Diabetes was defined as fasting plasma glucose (FPG)≥ 7.0 mmol/l (126 mg/dl) and/or a self-reported previous diagnosis established by health professionals (17).Hyperlipidemia was defined as high total cholesterol (TC) ≥ 6.2 mmol/L, and/or high triglycerides (TG) ≥ 2.3 mmol/L, and/or low high-density lipoprotein (HDL)< 1.0 mmol/L, and/or high low-density lipoprotein (LDL)≥ 4.1 mmol/L (18). Self-reported health was assessed by the question ‘In your opinion, what is your current state of health?’. The question was assessed and responses were categorised as “Bad”, “Ordinary” and “Good”. The third type was daily lifestyles, including sedentary behaviour. Sedentary behaviour was defined as prolonged sitting, reclining or lying in the waking state with an energy expenditure of no more than 1.5 metabolic equivalents. We classified sedentary time further into ≤3 hours and >3 hours based on previous literature (19).

### 2.3 Statistical analysis

In this study, independent t-test was used to test gender differences in the means of the variables, and Pearson *χ^2^* test was used to test the differences in proportions of variables. Considering the fact that mental health was a continuous variable, we used multiple linear regression model to analyse the effect of sleep duration on the mental health of middle-aged and older adults. Finally, Oaxaca-Blinder decomposition for linear model was used to describe the mental health difference between male and female middle-aged and older adults.

The Oaxaca-Blinder decomposition is a statistical method used to explain and quantify the difference in a certain indicator between two different groups (20, 21). This method can decompose the average difference between two different groups into two parts: the explained component and the unexplained component. The explained component represents the differences from the magnitude of the independent variables in the regression models, while the unexplained component is the result of different responses of the dependent variable to the independent variables. In this study, the Oaxaca-Blinder decomposition was used to decompose the mental health disparities of middle-aged and older people of different genders, and the decomposed coefficients and percentages were used to determine the strength of the impact of sleep duration on the mental health disparities of middle-aged and older people of different genders. Based on the hypothesis that “if middle-aged and older male adults had the same characteristics as their female counterparts, what would their mental health be?”, the mental health disparities between male and female groups are decomposed as follows:


$$\text{d}\text{i}\text{s}=E\left(Y_{u}-Y_{r}\right)=E\left[X_{u}\right]'\left(\beta_{u}-\beta_{r}\right)+\left(E\left[X_{u}\right]-E\left[X_{r}\right]\right)'\beta_{r}$$

where dis is the disparity of mental health among middle-aged and older people of different genders, u stands for female middle-aged and older people, r stands for male middle-aged and older people, Y stands for mental health, X indicates the independent variables, and indicates the estimated coefficients. $E\left[X_{u}\right]'\left(\beta_{u}-\beta_{r}\right)$ is the unexplained component, and $\left(E\left[X_{u}\right]-E\left[X_{r}\right]\right)'\beta_{r}$ is the explained component, which indicates differences in mental health due to individual characteristics.

In this study, SAS 9.4 software was employed to conduct descriptive statistics, T test, *χ* ^2^ test and multiple linear regression. Stata SE 15.1 software was employed to conduct Oaxaca-Blinder decomposition. All tests were two-sided and a *p*-value <0.05 was considered to be significant.

### 2.4 Ethical considerations

Our study was approved by the Biomedical Committee of Anhui Medical University (No. 832203441) and conducted in accordance with the principles of the Declaration of Helsinki. All participants were fully informed of the purpose and procedure of the study prior to participation and provided written informed consent. Participation was entirely voluntary, and respondents were authorised to withdraw at any time without any consequences. All data were collected anonymously using coded questionnaires, and only the research team had access to the raw data.

## 3 Results

### 3.1 Characteristics of participants

A total of 5–743 participants were involved in this study, with approximately equal proportions of males (49.02%) and females (50.98%). Less than half (49.50%) had a sleep duration ranging 7–8 hours per day. Regarding the socio-demographic characteristics, middle-aged and older people accounted for 74.16% and 25.84%, respectively. More than half (56.71%) participants had received more than six years of education, and only 5.07% had a marital status of widowed, divorced or unmarried. Regarding the physical health status, the prevalence rates of diabetes, hypertension, and hyperlipidemia were 15.72%, 27.67%, and 36.32%, respectively, and 50.72% self-reported average or poor physical health status. Regarding daily lifestyles, 42.59% of participants sat for more than three hours everyday (**Table 1**).

**Table 1:** Descriptive statistics.

Variable	Male (n=2 815)	Female (n=2 928)	All (n=5 743)	T test/*χ^2^* test
K10 score				<0.001
Mean (SD)	12.16 (4.19)	12.53 (4.31)	12.35 (4.25)	
Age				<0.001
<65	1 994 (70.83)	2 265 (77.36)	4 259 (74.16)	
≥55	821 (29.17)	663 (22.64)	1 484 (25.84)	
Education level				<0.001
≤0 yeas	998 (35.45)	1 488 (50.82)	2 486 (43.29)	
>6 yeas	1 817 (64.55)	1 440 (49.18)	4 357 (56.71)	
Marital status				<0.001
Widowed/divorced/separated	108 (3.84)	183 (6.25)	291 (5.07)	
Married	2 707 (96.16)	2 745 (93.75)	5 452 (94.93)	
Hypertension				<0.001
No	1 966 (30.16)	2 188 (74.73)	2 870 (72.33)	
Yes	849 (69.84)	740 (25.27)	2107 (27.67)	
Diabetes				0.035
No	2 343 (83.23)	2 497 (85.28)	4 840 (84.28)	
Yes	472 (16.77)	431 (14.72)	903 (15.72)	
Hyperlipidemia				0.089
No	1 824 (64.80)	1 833 (49.90)	3 657 (63.68)	
Yes	991 (35.20)	1 095 (37.40)	2 086 (36.32)	
Self-health				0.014
Bad	115 (4.09)	133 (4.54)	248 (4.32)	
Ordinary	1 299 (46.15)	1 366 (46.65)	2 665 (46.40)	
Good	1 401 (49.77)	1 429 (48.80)	2 830 (49.28)	
Sedentary behaviour				0.005
≤.0	1 563 (55.52)	1 734 (59.22)	3 297 (57.41)	
>3h	1 252 (44.48)	1 194 (40.78)	2 446 (42.59)	
Sleep time				<0.001
<7h	993 (35.28)	1 167 (39.86)	2 106 (37.61)	
7-8h	1 424 (50.59)	1 419 (48.46)	2 843 (49.50)	
>8h	398 (14.14)	342 (11.68)	740 (12.89)	

### 3.2 Bivariate analyses

The mental health score of middle-aged and older adults was 12.35 ± 4.25. There were significant differences in mental health between middle-aged and older males and females (*p*<0.001). Specifically, the average score of middle-aged and older females was significantly higher than that of males. In addition, significant differences were observed between males and females in terms of basic demographic characteristics, sleep duration, sedentary behaviour, prevalence of chronic diseases, and self-rated health status (*p*<0.05) (**Table 1**).

### 3.3 Multiple regression analysis

The results of multiple regression analysis showed a significant negative correlation between sleep duration and K10 scores in male (*β*=-0.404, *p*<0.05) and female middle-aged and older adults (*β*=-0.420, *p*<0.05). Moreover, sleep duration had a stronger effect on the mental health of middle-aged and older females compared to males. Hypertension and sedentary behaviour (*p*<0.05) were associated with poorer mental health. However, participants who had higher levels of education, were married and self-reported bad health were less likely to have mental health problems (*p*<0.05). In addition, age and dyslipidaemia were only statistically significant for mental health status in female participants (**Table 2**).

**Table 2:** Regression result of the effect of sedentary behaviour on psychological health.

Variable	Male (n=2 815)	Female (n=2 928)
Age	0.308 (0.173)	-0.677*** (0.188)
Education level	-0.547** (0.168)	-0.585*** (0.160)
Marital status	-0.989* (0.400)	-1.782*** (0.315)
Hypertension	0.721*** (0.171)	0.753*** (0.178)
Diabetes	0.148 (0.207)	0.028 (0.217)
Hyperlipidemia	0.132 (0.163)	0.572*** (0.158)
Self-health	-0.721*** (0.171)	-0.753*** (0.178)
Sedentary behaviour	0.320* (0.154)	0.388* (0.157)
Sleep time	-0.404*** (0.113)	-0.420*** (0.115)
Constant	18.252*** (0.933)	21.559*** (0.827)

Robust standard errors are given in parentheses. ^*^ *p*<0.05; ^**^ *p*<0.01; ^***^ *p*<0.001.

### 3.4 Oaxaca–Blinder decomposition analysis

**Table 3** showed the Oaxaca-Blinder decomposition results for the impact of sleep duration on the mental health disparity among middle-aged and older people of different genders. The mental health disparity between middle-aged and older male and female was -0.3699, with the explained part and the unexplained part being -0.1400 and -0.2300, accounting for 37.85% and 62.18% of the total difference, respectively. In the explained part, sleep duration (20.57%) made a significant contribution to the mental health difference between middle-aged and older people of different genders. Additionally, the differences in educational level, marital status, hypertension prevalence, and sedentary behaviour between middle-aged and older male and female contributed to the mental health differences by 61.86%, 24.57%, -24.57%, and -9.36%, respectively.

**Table 3:** Oaxaca-Blinder decomposition result of the effect of sedentary behaviour on male-female psychological health disparity.

Variable	Coefficient	Robust standard error	Contribution (%)^a^
Male	12.1581	0.079	
Female	12.5280	0.080	
Difference	-0.3699^**^	0.112	100%
Explained	-0.1400^**^	0.041	37.85%
Unexplained	-0.2300^*^	0.108	62.18%
Age	-0.0087	0.009	6.21%
Education level	-0.0866^***^	0.020	61.86%
Marital status	-0.0344^**^	0.012	24.57%
Hypertension	0.0344^**^	0.011	-24.57%
Diabetes	0.0014	0.003	-1.00%
Hyperlipidemia	-0.0077	0.005	5.50%
Self-health	-0.0227	0.024	16.21%
Sedentary behaviour	0.0131^*^	0.006	-9.36%
Sleep time	-0.0288^*^	0.009	20.57%

^*^ *p*<0.05; ^**^ *p*<0.01; ^***^ *p*<0.001. ^a^Percentage contribution to the explained differences in gender disparities in mental health. The Oaxaca-Blinder decomposition method ensures that the sum of the contributions of all control variables in the estimation equation equals the explained differences.

## 4 Discussion

This study surveyed the mental health status of 5–743 middle-aged and older adults aged 45 and above, and analysed the impact of sleep duration on the mental health of different gender. The results showed that the Kessler10 score for middle-aged and older adults in central China was (12.35 124.25). Among them, female middle-aged and older adults had relatively high mental health scores, indicating worse mental health conditions. This was similar to previous studies (22, 23). For example, a survey in South Australia found that the average mental health score for older people aged 60 and above was 12.5, with the level of psychological distress being significantly higher in female than in male (22). A survey conducted by Yang et al. on older people of Chongqing found that the prevalence of psychological distress was significantly higher in female (11.60%) compared to male (8.90%) (23). Our results suggested that health departments should focus more on the mental health status of middle-aged and older female during mental health prevention and treatment processes.

The study found that there was a significant association between sleep duration and mental health status of middle-aged and older adults. Additionally, The correlation between sleep duration and the mental health of middle-aged and older women is stronger than that of men. This had also been confirmed in previous studies (24). Gender differences in the relationship between sleep duration and mental health in middle-aged and older adults might be related to differences in physiological structure between males and females (25). A review study on the relationship between sleep and sex hormones in adult female showed that female gradually experience menopause and perimenopause after the age of 45, during which dramatic changes in oestrogen levels might make middle-aged and older female more sensitive to changes in sleep quality and duration, adversely affecting their daily emotions (24). Besides, the division of gender roles in society might also have some relevance to mental health differences. With the intensification of aging in China and continuous adjustments to birth policies, intergenerational care had become a common social phenomenon. Under the traditional family division of labour “men were breadwinners, women were homemakers,” middle-aged and older women participated in intergenerational care at a much higher rate than men. A study by Zhao et al. on the mental health status in China showed that the effort put in by grandmothers during caregiving for grandchildren not only increased their time pressure and sleep deprivation but also significantly raised the risk of depressive symptoms (26). This suggests that sleep interventions targeting middle-aged and older women should be a key entry point for narrowing the gender gap in mental health. In the future, sleep health assessments and management should be incorporated into routine health check-ups in conjunction with menopause care services, and targeted sleep hygiene education (such as regular schedules and creating a suitable sleep environment) should be provided to improve sleep habits. At the same time, attention should be paid to the social role pressures they face, and community support services should be explored and developed to reduce their caregiving burden, thereby creating favourable conditions for improving sleep quality and duration.

Furthermore, our study found that gender differences in sleep duration explained 20.57% of the total variation in mental health among middle-aged and older adults. If the sleep duration of middle-aged and older male were the same as that of female, the difference in mental health could be reduced by about 20.57%. A survey by Boerma et al. of 59 countries showed that health inequality by gender was a common phenomenon, with women’s sleep quality and psychological status scores being significantly lower than those of men (27). Sleep is an important predictor of mental health issues. Chronic sleep deprivation could damage the neurobiological mechanisms related to the sleep-wake cycle, affecting emotional stability and exacerbating negative emotions (28, 29). Therefore, when formulating mental health prevention and treatment strategies for middle-aged and older people, priority should be given to narrowing the gender gap in sleep among middle-aged and older people, and this should be regarded as key to improving overall mental health and reducing gender inequality.

Mental health is a comprehensive concept, which is closely related to a variety of factors such as individual characteristics, lifestyle and social environment. This study found that in addition to sleep duration, educational level, marital status, and hypertension prevalence and sedentary behaviour were also important explanatory components of the differences in mental health between male and female middle-aged and older adults. Social withdrawal theory (30) suggests that sedentary behaviour reduces the frequency of social interaction and social support among middle-aged and older adults, leading to a range of mental health problems. A study that included 67,077 residents from 30 countries showed that among those who were sedentary for >8 h per day, women had a significantly higher risk of developing depressive symptoms than men (31). Therefore, a multilevel support system needs to be constructed to improve the mental health of middle-aged and older adults by implementing differentiated strategies in chronic disease management, social role adjustment and sedentary behavioural interventions are required.

This study conducted an investigation into the mental health and related factors among middle-aged and older adults of different genders in central China, and used the Oaxaca-Blinder decomposition method to explore the impact of sleep duration on the mental health in middle-aged and older adults of different genders. The research results filled the gap regarding gender differences in the impact of sleep duration on the mental health of middle-aged and older adults in China. However, there were some limitations to this study. Firstly, due to the cross-sectional survey design of this study, we only explored the correlation between sleep duration and mental health of residents of different genders, and could not accurately determine the causal relationship between them. Second, this study used the Oaxaca-Blinder model to assume a linear relationship and ignored the interaction between variables, which may have underestimated the synergistic effects of sleep duration and other factors (such as chronic diseases). Third, due to data limitations, this study only focused on gender differences in the relationship between sleep duration and mental health, and did not include other sleep indicators such as sleep efficiency, sleep latency, and daytime dysfunction, as well as did not take into account the effects of gender-specific variables such as retirement roles and marital/family responsibilities on mental health differences. Future studies need to incorporate different dimensions of sleep and gender-related factors to reveal the gender heterogeneity of the relationship between sleep and mental health.

## 5 Conclusion

In summary, the results of this study indicated significant gender differences in the mental health status of middle-aged and older adults, and female’s mental health was poorer compared to that of male. Additionally, differences in sleep duration are an important component of the mental health gap between middle-aged and older people of different genders. These findings provide key evidence from China on the widespread phenomenon of gender inequality in health, clearly demonstrating the critical role of improving sleep quality among older women in narrowing the gender gap in mental health and enhancing the overall mental health of the population. In the future, targeted prevention and intervention measures should be taken based on the gender differences in sleep duration and mental health among middle-aged and older adults, including the development of personalised sleep management plans and regular sleep quality assessments, in order to decrease the risk of mental health problems.

## Data Availability

The raw data supporting the conclusions of this article will be made available by the authors, without undue reservation.
